# A Sensitivity Analysis of the Impact of Rain on Regional and Global Sea-Air Fluxes of CO_2_

**DOI:** 10.1371/journal.pone.0161105

**Published:** 2016-09-27

**Authors:** I. G. Ashton, J. D. Shutler, P. E. Land, D. K. Woolf, G. D. Quartly

**Affiliations:** 1 Centre for Geography, Society and the Environment, University of Exeter, Penryn Campus, Cornwall, TR10 9EZ, United Kingdom; 2 Plymouth Marine Laboratory, Prospect Place, West Hoe, Plymouth, PL1 3DH, United Kingdom; 3 International Centre for Island Technology, Stromness, Orkney, KW16 3AW, United Kingdom; University of Vigo, SPAIN

## Abstract

The global oceans are considered a major sink of atmospheric carbon dioxide (CO_2_). Rain is known to alter the physical and chemical conditions at the sea surface, and thus influence the transfer of CO_2_ between the ocean and atmosphere. It can influence gas exchange through enhanced gas transfer velocity, the direct export of carbon from the atmosphere to the ocean, by altering the sea skin temperature, and through surface layer dilution. However, to date, very few studies quantifying these effects on global net sea-air fluxes exist. Here, we include terms for the enhanced gas transfer velocity and the direct export of carbon in calculations of the global net sea-air fluxes, using a 7-year time series of monthly global climate quality satellite remote sensing observations, model and in-situ data. The use of a non-linear relationship between the effects of rain and wind significantly reduces the estimated impact of rain-induced surface turbulence on the rate of sea-air gas transfer, when compared to a linear relationship. Nevertheless, globally, the rain enhanced gas transfer and rain induced direct export increase the estimated annual oceanic integrated net sink of CO_2_ by up to 6%. Regionally, the variations can be larger, with rain increasing the estimated annual net sink in the Pacific Ocean by up to 15% and altering monthly net flux by > ± 50%. Based on these analyses, the impacts of rain should be included in the uncertainty analysis of studies that estimate net sea-air fluxes of CO_2_ as the rain can have a considerable impact, dependent upon the region and timescale.

## Introduction

The sea-air exchange of the greenhouse gas carbon dioxide (CO_2_) is a critical part of the climate system and a major factor in the biogeochemical development of the oceans. It is widely accepted that more accurate and higher resolution calculations of these gas exchanges (fluxes) are required if we are to fully understand and predict our future climate. Such knowledge is also required for understanding and monitoring chemical water quality (e.g. in relation to ocean acidification).

The impact of raindrops falling on the sea can influence the rate of gas transfer between the ocean and the atmosphere by increasing surface turbulence. The addition of rainwater will change the temperature, salinity and carbonate composition of surface waters, affecting the solubility and partial pressure of CO_2_ (*pCO*_2_) in the surface layer. Rain will also directly transfer dissolved CO_2_ to the ocean (termed wet deposition).

Early work by Ho et al. [1] highlighted how rain can significantly enhance gas transfer and provided the first parameterisation of rain-driven gas transfer velocity for freshwater environments. This work was extended towards determining the physical mechanisms underlying this enhancement, showing that the impact of rain in freshwater systems caused surface bubbles and waves and that the enhancement of gas transfer was mainly due to an increase in surface turbulence [2]. It was subsequently shown that the rain-driven gas transfer velocity was similar for freshwater and saltwater systems, although differences in vertical mixing in the saltwater system due to stratification meant that gas flux in the seawater system was lower [3]. More recently, Zappa et al. [4] showed that rain-induced turbulence was the main reason for rain enhanced gas transfer in saltwater systems. They also showed that the gas transfer velocity scaled with the turbulent dissipation rate.

Rain and wind effects were initially understood to combine linearly to influence gas transfer velocity, *k* [3]. However, Harrison et al. [5] showed that whilst rain can contribute significantly to the total sea-air gas flux at low wind speeds, at higher wind speeds the effects become negligible and a new non-linear parameterisation for the gas transfer in field conditions was presented [5].

The changes in temperature, salinity and carbonate composition of surface waters caused by the introduction of rainwater will alter the solubility and partial pressure of CO_2_ (*pCO*_2_) in the surface layer. Data sets gathered from in-situ or satellite remote sensing are selected or adjusted to represent the surface water conditions that dominate sea-air gas flux [6]. On the spatial and temporal scales resolved by these data, stratification and the influence of rain on surface waters will be represented and thus accounted for in exchange calculations. However, rain events will occur at spatial and temporal scales that are not resolved by the data used for these global studies, such as short, intense rain showers. As such, the temporary dilution of surface water during rain events, that has the potential to affect the sea-air gas flux, will not be resolved in this analysis.

Dilution effects have received less research attention to date when compared with the enhancement of gas transfer due to rain, although Turk et al. [7] provide initial experimental evidence that dilution affects regional sea-air CO_2_ flux. Salinity gradients in the top few meters of the ocean surface due to freshwater input from rain have been studied for their influence on remotely sensed salinity measurements [8], and can be related to rain rate [9]. Santos-Garcia et al. [10] present a physics-based model that draws on very high resolution modeled precipitation estimates (NOAA CMORPH) and surface wind data in order to predict surface stratification due to rain. However, the spatial and temporal resolution required to resolve individual rain events is not currently compatible with the global data sets used in the calculations presented here.

Previous studies of the type presented here include Komori et al. [11], who accounted for enhanced transfer velocity using results from their laboratory tests and direct wet-deposition. They estimated that the global effect of rainfall on net sea-air fluxes for the year 2001 was to increase the sink of atmospheric CO_2_ by <5%. Following this, Turk et al. [7] incorporated laboratory-derived parameterisations of wet deposition, rain-enhanced *k* and surface *pCO*_2_ dilution, into flux estimates for a single location in the Western Equatorial Pacific. When extrapolated across the region, these point values indicated an increased uptake of CO_2_, with the net flux in the Western Equatorial Pacific Ocean changing from a source to a sink. The findings highlight the significant role that rain can play, particularly in regions characterised by low winds and high precipitation and support the need for the global and regional impact of rain to be considered in gas flux studies.

In synopsis, previous work studying the impact of rain on sea-air fluxes of CO_2_ has focussed on laboratory studies [1–3, 5, 12], localised field studies, including the Biosphere 2 model ocean [1, 4, 12], and the use of one-dimensional numerical-models [3, 4, 7]. Regional and global estimates of integrated net sea-air fluxes have largely ignored the impact that rain can have, and most global studies do not account for rain within their uncertainty analyses. The exception to this is the work of Komori et al. [11] who applied laboratory-derived parameterisations to study global sea-air fluxes for a single year (2001) using model, climatology and Global Precipitation Climatology Project (GPCP) data.

The FluxEngine software tool offers an efficient mechanism to exploit up to 20 years of Earth observation (EO) and blended EO and in situ data, in order to calculate global and regional estimations of sea-air CO_2_ flux [6]. Global gas exchange requires a large and complex set of calculations. Variations or errors in these calculations can hinder intercomparison between studies and are difficult to identify without interrogating actual calculations procedures. The FluxEngine has been created to provide a consistent set of calculations that reduce the repeated effort required for studies in this field. It has been extensively verified against known datasets to provide a common baseline for the international community, such that its use minimises errors and helps maintain consistent analysis between studies. The software tool and associated publications are open access and can be accessed through the project website (www.oceanflux-ghg.org). The source code is also open source. It is continually updated to keep up with advances in the field and can be downloaded here, github.com/oceanflux-ghg/FluxEngine.

The work in this paper uses FluxEngine to build upon and extend the work of Komori et al. [11] by applying recent advances, parameterisations and tools in order to characterise the potential global and regional impacts that rain can have on the different components of the sea-air flux calculation. The components considered are rain-induced gas transfer velocity and the direct wet deposition of CO_2_ by raindrops landing on the ocean surface. Results are presented as monthly and annual net fluxes for global and regional seas, providing an inter-annual and seasonal assessment of the net impact of rain on global flux of CO_2_. These estimates are driven by two different CO_2_ climatologies, that presented by Takahashi et al. [13], and that provided by SOCAT [14]. These climatologies are referenced to single years, 2000 and 2010 respectively. As such, inter-annual variability is estimated solely through changes in sea surface temperature (SST), wind and rainfall, and does not reflect changes in pCO_2w_. Inter-annual results are analysed in terms of the sensitivity of the global and regional estimates to rain, identifying regions where rain can have a significant impact on sea-air CO_2_ gas exchange, whilst acknowledging the unknown effect of changes in pCO_2w_. The final part of the paper includes a discussion of the impact of rain-driven dilution of the surface layer, including an initial analysis of the impact of rain-driven variations in the sea skin temperature (SST_skin_).

## Methods

The global impact of rain on sea-air CO_2_ fluxes is studied using monthly, multi-year data. The following sections describe the datasets used as well as the methods for calculating the monthly sea-air CO_2_ fluxes, the rain-driven gas transfer velocity and the wet deposition of CO_2_. Calculations were undertaken using the FluxEngine open source processing toolbox [6]. This toolbox allows users to easily parameterize and generate global and regional sea-air CO_2_ flux estimates. For this study the toolbox was extended to allow rain induced transfer and wet deposition to be included in the air-sea gas flux parameterisation. Here we study the four major ocean basins, Atlantic, Indian, Pacific and Southern. Detailed definitions of these regions, verification of the system and the range of configurations available are presented in Shutler et al. [6].

### 2.1 Datasets

To characterise the sea surface, we first used satellite EO data from the European Space Agency (ESA) Sea Surface Temperature Climate Change Initiative data (version 1.1.1) for *SST*_*skin*_, (K) [15] and ESA GlobWave for wind speed at 10 m, *U*_10_ (m s^-1^) [16]. Both of these datasets are calibrated, bias corrected, well-characterised with known uncertainties and designed for use in climate studies. For ice cover we use satellite based, Special Sensor Microwave Imager (SSM/I) global percentage ice cover data [17, 18]. These datasets have been re-gridded onto a 1° × 1° grid where each grid value was the statistical mean of all contributing data [6]. For surface salinity (S), we use the World Ocean Atlas salinity data provided within Takahashi et al. [13]. For in-water *pCO*_2_ (*pCO*_2*W*_) we use two different data sets. Firstly, the climatological data from Takahashi et al. [13] with a reference year 2000 and an estimated global increase in *p*CO_*2w*_ of 1.5 μatm yr^-1^ (eq 5). Moving further away from this reference year, the estimated temporal correction for the CO_2_ climatology becomes less robust. Thus, the study using [13] was limited to the years 1999–2006, where the correction is most appropriate. The flux estimates are expected to be strongly dependent on the accuracy of the *pCO*_*2W*_ climatological data. In order to examine this sensitivity, an alternative climatological *pCO*_*2w*_ dataset was also used, which is derived from the SurfaceOcean CO_2_ Atlas (SOCAT) [14]. Notably here, the reference year is 2010. The timescales between these two *pCO*_2*W*_ data sets do not match, but they do overlap, allowing the impact of the choice of *pCO*_2*W*_ dataset to be determined.

We calculate atmospheric *pCO*_2_ (*pCO*_2*A*_) using modelled air pressure (*P*) and climatological concentration of CO_2_ in dry air (*X*_*CO*2*A*_) from the NCEP CFSR model [13]. The *pCO*_2*W*_, *P* and *X*_*CO*2*A*_ data were linearly interpolated to the same 1° × 1° grid as the other datasets. For rain rate we used the daily 1° × 1° GPCP, version 2.2 [19]. There is still considerable debate about the absolute magnitudes of the global distribution of precipitation and its seasonal variation [20, 21], although the GPCP dataset is widely accepted as one of the most reliable.

### 2.2 Sea-air CO_2_ flux

The sea-air flux of CO_2_ (*F*, g m^-2^ s^-1^), is calculated using the product of a gas transfer velocity, *k* (m s^-1^), and the difference in CO_2_ concentration (g m^-3^) between the base [*CO*_*2AQW*_] and the top [*CO*_*2AQ0*_] of a thin (~10 to 250 μm) boundary layer at the sea surface:

$$F=k\left(\left[CO_{2AQW}\right]-\left[CO_{2AQ0}\right]\right)$$ (1)

The concentration of CO_2_ in seawater is the product of its solubility, *α* (g m^-3^ μatm^-1^), and its fugacity, *fCO*_2_ (in μatm). Gas solubility is a function of salinity and temperature and as such, it varies across the aqueous boundary layer. (Eq 1) then becomes:

$$F=k\left(\alpha_{W}fCO_{2W}-\alpha_{S}fCO_{2A}\right)$$ (2)

where the subscripts denote values in water (*W*), at the sea-air interface (*S*) and in air (*A*). For simplicity we can substitute partial pressure for fugacity because their values differ by <0.5% over the temperature range considered [22]. Therefore we estimate the sea-air flux using:

$$F=k\left(\alpha_{W}pCO_{2W}-\alpha_{S}pCO_{2A}\right)$$ (3)

Climatological estimates of *pCO*_2*W*_ (*pCO*_2*Wclim*_) must be adjusted to the SST for the period of study. Following previous studies [23–25], the *pCO*_2*W*_ values were corrected to reflect SST using the relationship provided by Takahashi et al. [13]:

$$pCO_{2W}=pCO_{2Wclim}\left(exp\left(0.0423\left(SST-T_{clim}\right)\right)-4.35\times 10^{-5}\left[SST^{2}-T_{clim}^{2}\right]\right)$$ (4)

where *T*_*clim*_ is the temperature from the Takahashi et al. [13] climatology in °C, and *SST* is estimated as *SST*_*skin*_ + 0.17 and converted to °C [26].

*pCO*_2*A*_ (in μatm) was calculated by including a global average increase of 1.5 μatm yr^—1^ using: [13]

$$pCO_{2A}=x_{CO2A}\left(P-pH_{2}O\right)+1.5\left(y-2000\right)$$ (5)

where *y* is the year, *P* is the daily average air pressure (in μatm), *X*_*CO*2*A*_ is the zonal mean molar fraction of CO_2_ in the dry atmosphere (in parts per million) and *pH*_2_*O* is the saturation vapour pressure in μatm [27]:

$$pH_{2}O=1013.25exp\left[24.45-\left(67.45\left(\frac{100}{SSTK}\right)\right)-\left(4.85\text{ ln}\left(\frac{SSTK}{100}\right)\right)-0.00054\text{S}\right]$$ (6)

where salinity, *S* is on the Practical Salinity Scale and air temperature, and *SST*_*k*_ is subskin sea surface temperature in Kelvin.

### 2.3 Rain impacts

The sea-air flux due solely to wet deposition is estimated using [11]:

$$F_{DIC}=\;-Rn\;\alpha \;pCO_{2A}$$ (7)

where *Rn* is the rain rate in mm h^-1^ and *α* is the solubility of CO_2_ in fresh water, calculated for local air temperature, but with salinity set to 0, using the formulation in Wanninkhof [28].

Initial laboratory experiments derived a linear increase in the transfer velocity during rain events, dependent on *Rn*, [1]

$$k_{total}=k_{wind}+k_{rain}$$ (8)

where,

$$k_{rain}=\;\left(0.929\;+\;0.679\;Rn\;–\;0.0015\;Rn_{2}\right)$$ (9)

Recent work has shown how the rain influences the gas transfer velocity in a nonlinear fashion [5]. Therefore, the total gas transfer velocity (*k*_*total*_) due to wind and rain is defined as:

$$k_{total}=k_{wind}++\left[1-\text{exp}\left(-\alpha \beta \right)\right]\;k_{rain}$$ (10)

where *a* = 0.3677 and *β* = *KEF*_*r*_ */ KEF*_*w*_, where *KEF*_*r*_ is the kinetic energy flux due to rain, and *KEF*_*w*_ is that imparted to the water by surface winds. Harrison et al. [5] assume a Laws-Parsons raindrop-size distribution to derive a simplified relationship, *KEF*_*r*_ = 0.0112*Rn* and define *KEF*_*w*_ = *ρ*_*a*_ u*^3^, where *ρ*_*a*_ is the density of air (in kg m^-3^) defined as *ρ*_*a*_ *= P /(R SST*_*k*_*)*, where *P*, is he air pressure (in Pa) and *R* is the specific gas constant for dry air (in J kg^-1^ K^-1^). The friction velocity u* (in ms^-1^) is given by u*^2^ *= C*_*D*_ *U*_10_^2^, where *C*_*D*_ is the drag coefficient as defined by Yelland and Taylor [29].

The wind speed parameterised gas transfer velocity, *k*_*wind*_, was estimated following the method in [13] such that, *k*_*wind*_ = 0.26(*U*_*10*_)^2^ (*Sc*/660)^-1/2^, where *Sc* represents the Schmidt number of the gas in question. *k*_*wind*_ was used to calculate a reference flux, *F*_*ref*_, in which no contribution from rain was included:

$$F_{ref}=k_{wind}\left(\alpha_{W}\text{pC}O_{2W}-\alpha_{S}\text{pC}O_{2A}\right)$$ (11)

The [1*-* exp(*-aβ*)] *k*_*rain*_ term in (eq 10) represents the enhancement of the gas transfer velocity due to rain rate through a non-linear relationship with wind speed. The combined wind and rain sea-air CO_2_ flux is then given by:

$$F_{k-rain}=k_{total}\alpha_{W}\left(\text{pC}O_{2W}-\text{pC}O_{2A}\right)$$ (12)

The total sea-air flux, *F*_*T*_, that includes the rain impacts described above is then the sum of the gas transfer and the wet deposition components:

$$F_{T}=F_{k-rain}+F_{DIC}$$ (13)

The contributions from rain effects were then calculated as the difference between the rain affected flux values, (*F*_*T*_, *F*_*k-rain*_ and *F*_*DIC*_) and *F*_*ref*_. In this study, *F* values represent the sea-air CO_2_ flux. Positive values represent an outgassing of CO_2_ from the ocean to the atmosphere, whilst negative values represent a transfer (sink) of CO_2_ from the atmosphere to the oceans.

### 2.4 Integrated net sea-air fluxes

Integrated fluxes over a given region are calculated from the monthly mean flux at each pixel, adjusted for ice and the pixel's total area, which is calculated assuming the Earth to be an oblate spheroid. Missing data values are accounted for using a regional average and added to the integrated net flux from valid data values, to give an estimate of the total regional integrated net flux [6]. Global values are estimated by treating the entire globe as a single region. We refer the reader to the [6] for a detailed description of the integrated net flux tool which is part of the FluxEngine.

### 2.5 Uncertainties

An ensemble approach was adopted to assess the uncertainties in *F*_*T*_. Random errors were used to perturb input data for multiple runs, according to known variability in the input data sets. Uncertainties in the rain data set are provided through the GPCP [19] as a variance for each datum, *σ*_*i*_^2^, which includes both algorithm and random sampling errors that can vary in time and space. Bias is considered to be zero [19]. Using the values presented in Land et al. [25], the variabilities of *U*_10_, *SST* and *pCO*_2W_ were estimated as published global standard deviations that do not vary in time or space.

Following the method used by Land et al. [25], a random noise signal was generated for each parameter and used to perturb the input data. For rain rates, noise was added by using a value drawn at random from a normal distribution with mean *X*_*i*_ equal to the original value and standard deviation *σ*_*i*_ equal to the uncertainty value provided, N(*X*_*i*_, *σ*_*i*_). Resulting rain rates less than zero were set to 0. For *U*_10_, *SST* and *pCO*_2W_, noise was added by using a value drawn at random from a log-normal distribution, with a (natural) log mean equal to the log of the original data point and the published log standard deviation, exp [N(ln *X*_*i*_, *σ*)].

This process was repeated with 10 different perturbations for the year 2000, producing 10 separate sets of monthly and annual results. The uncertainty estimates provided here are the standard deviation of results across these 10 runs. The cumulative effect of the uncertainties on each parameter was used as an indication of total uncertainty in the resulting flux. This assumes that all of the errors are uncorrelated. In reality, there will be some inter-dependence between the input parameters, which will affect the stated errors in CO_2_ flux.

## Results

The following sections present the results for the global oceans and the individual oceanic basins.

### 3.1 Annual integrated net sea-air CO_2_ fluxes

**Fig 1:**
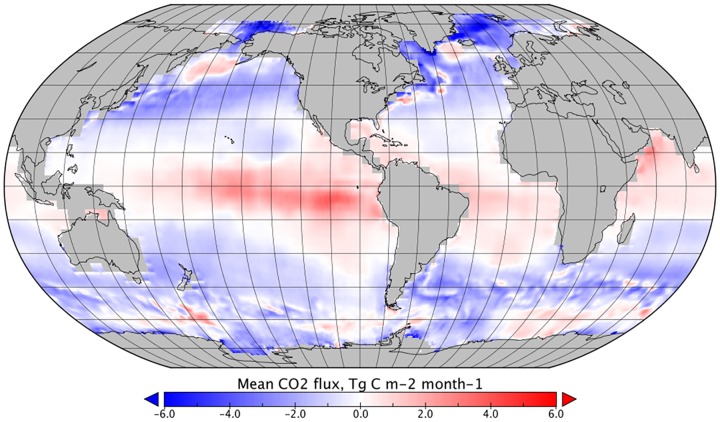
Mean monthly CO_2_ flux between January 1999 and December 2005 for a reference dataset (no rain components).

**Fig 2:**
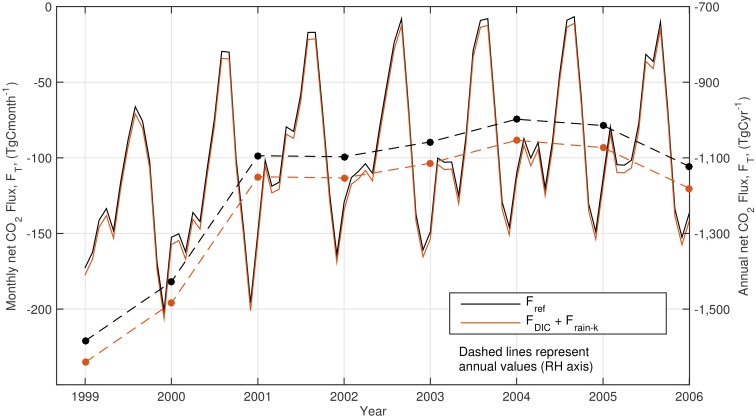
Annual (right axis, solid lines) and monthly (left axis, dashed lines) global net sea-air CO_2_ flux, without the effects of rain, *F*_*ref*_, and with the effects of rain, *F*_*T*_ = *F*_*DIC*_ *+ F*_*k-rain*_.

For the CO2 climatology reference year (2000), the estimated annual global sea-air CO_2_ flux without any rain impacts is -1.4 Pg C yr^-1^. The FluxEngine has been validated with previous outputs in this research field [6] and these annual net integrated values are consistent with the original publication of Takahashi et al. [13]. When the CO_2_ climatology was used to study subsequent years (eq 5), applying SST, wind and other data from each year, the annual values are consistently negative and between -1 Pg C yr^-1^ and -1.6 Pg C yr^-1^, i.e. the global ocean is a net sink of CO_2_ (Figs 1 and 2 and Table 1). Global estimates are significantly lower during the years 1999 and 2000, meaning the net sink of CO_2_ is at its greatest. Notably, these years correspond to a strong La Niña event.

**Table 1:** Annual global integrated net flux, *F*_*T*_ (Tg C yr^-1^) with and without rain (left hand columns) and the impact of each rain component on *F*_*T*_ (Tg C yr^-1^) (right hand columns), where *F*_*T*_ = *F*_*DIC*_ + *F*_*k-rain*,_ (non-linear).

	Net CO2 Flux, *F*, Tg C yr^-1^		Effect on CO2 flux, *ΔF*, Tg C yr^-1^			
	Reference, *F*_*ref*_	*F*_*T*_	*F*_*T*_	*F*_*DIC*_	*F*_*k-rain*_, linear	*F*_*k-rain*_, non-linear
1999	-1584.24	-1640.88	-56.64	-59.70	-27.76	3.06
2000	-1427.66	-1484.11	-56.45	-60.47	-18.29	4.03
2001	-1094.42	-1150.03	-55.61	-60.08	-2.45	4.47
2002	-1097.07	-1153.14	-56.07	-62.85	2.56	6.78
2003	-1057.83	-1114.33	-56.50	-62.40	4.27	5.90
2004	-997.91	-1053.65	-55.74	-62.19	3.96	6.45
2005	-1014.53	-1073.01	-58.48	-63.59	2.67	5.11
2006	-1122.76	-1180.59	-57.83	-63.67	2.13	5.84

**Fig 3:**
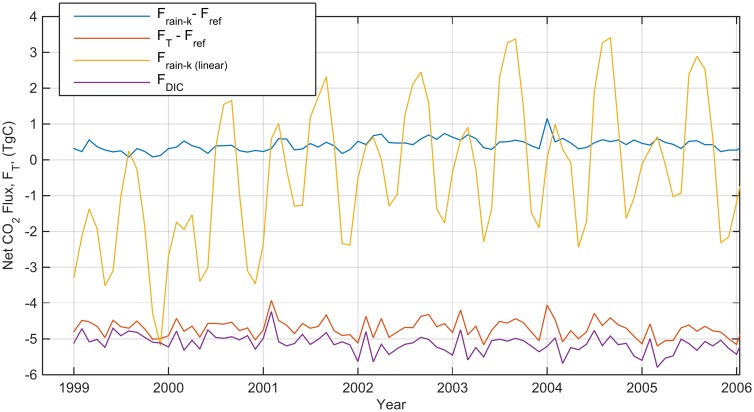
The monthly mean global CO_2_ flux attributed to the enhancement of transfer velocity (both non-linear, *F*_*rain-k*_ and non-linear, *F*_*rain-k (linear)*_) and Direct deposition, *F*_*DIC*_, TgC month^-1^.

**Fig 4:**
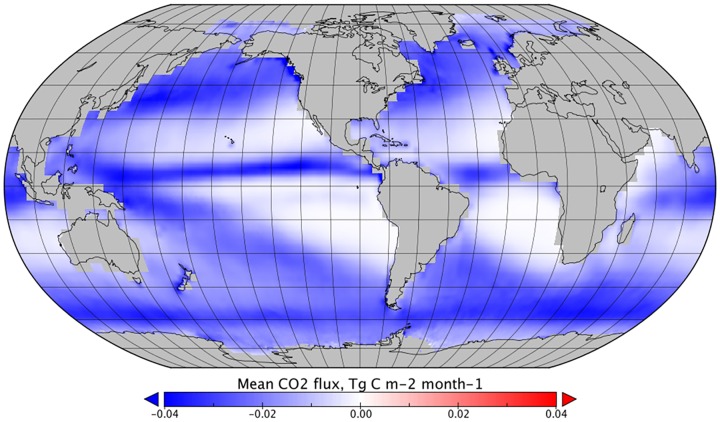
The mean effect of wet deposition on monthly CO_2_ flux between January 1999 and December 2005, *F*_*DIC*_*—F*_*ref*_.

**Fig 5:**
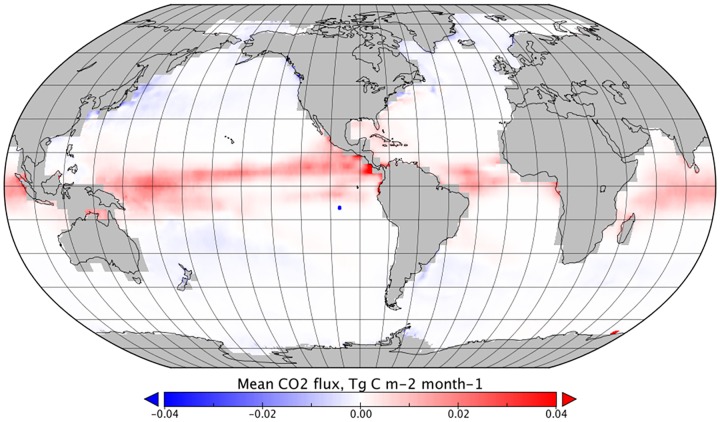
The effect of rain on monthly CO_2_ flux between January 1999 and December 2005, given a non-linear model of transfer velocity (eq 10), *F*_*k-rain*_*—F*_*ref*_.

**Fig 6:**
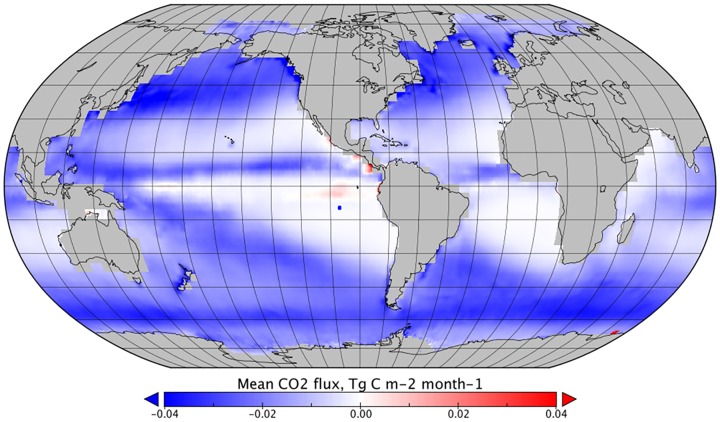
The combined effect of wet deposition and non-linear gas transfer velocity on CO_2_ flux between Jan 1999 and Dec 2005, (*F*_*DIC*_ + *F*_*k-rain*_) —*F*_*ref*_.

The change to global net sea-air flux due to direct wet deposition of CO_2_, *F*_*DIC*_, varies from -60 to -64 Tg C yr^-1^ (Figs 3 and 4 and Table 1). The effect of rain enhancing gas transfer velocity, for a non-linear model (eq 10), varies from 3 to 6 Tg C yr^-1^ (Figs 3 and 5 and Table 1). Assuming a linear sum of these components gives an effect on annual global net sea-air CO_2_ flux of -56 to -58 Tg C yr^-1^ (Figs 3 and 6 and Table 1). When compared to the estimated annual net integrated sea-air CO_2_ flux without any rain impacts, this equates to an increase in the global oceanic CO_2_ sink of 3.5 to 6%.

**Fig 7:**
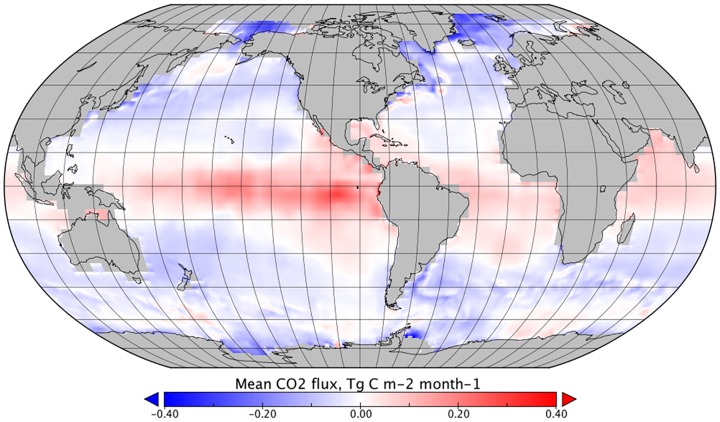
The mean effect of rain on monthly CO_2_ flux between January 1999 and December 2005, given a linear model (Ho 2004), *F*_*k-rain(linear)*_*—F*_*ref*_. Note different scale compared to Figs 4, 5 and 6.

Comparison of flux estimations between those made using a linear relationship between wind and rain (eq 8) and those made with a non-linear relationship (eq 10) showed notably different results. The non-linear parameterisation decreased the oceanic CO_2_ sink, whilst the linear parameterisation increased the oceanic CO_2_ sink (Figs 3 and 7). The linear parameterisation also exhibited seasonal variations with magnitude up to 10 times greater than the non-linear parameterisation (Fig 3 and Table 2). A similar effect was observed in average global flux, where the linear parameterisation showed significantly higher geographic variability, ranging from an average of -0.4 to 0.4 Tg C month^-1^, compared to -0.02 to 0.02 Tg C month^-1^ for the non-linear parameterisation. Following recommendations in Harrison et al. [5], the non-linear parameterisation was adopted and the remaining results in this paper that include *k*_*rain*_ or *F*_*k-rain*_ refer to the non-linear parameterisation (eq 10).

**Table 2:** Monthly global integrated net flux, *F*_*T*_ (Tg C yr^-1^) with and without rain (left hand columns) and the impact of each rain component on *F*_*T*_ (Tg C yr^-1^) (right hand columns), where *F*_*T*_ = *F*_*DIC*_ + *F*_*k-rain*_ (non-linear).

Month	Net CO2 Flux, *F* Tg C mnth^-1^		Effect on CO2 flux, *ΔF*, Tg C mnth^-1^			
	Reference, *F*_*ref*_	*F*_*T*_	*F*_*T*_	*F*_*DIC*_	*F*_*k-rain*_, linear	*F*_*k-rain*_, non-linear
Jan	-139.08	-143.92	-4.84	-5.33	-1.31	0.49
Feb	-114.47	-118.83	-4.37	-4.76	-0.14	0.40
Mar	-119.72	-124.62	-4.90	-5.49	0.00	0.59
Apr	-112.40	-117.11	-4.71	-5.21	-0.58	0.50
May	-117.18	-122.16	-4.97	-5.33	-2.09	0.35
Jun	-87.63	-92.30	-4.67	-4.99	-1.64	0.32
Jul	-53.22	-57.80	-4.58	-5.02	1.22	0.44
Aug	-27.04	-31.72	-4.68	-5.11	2.29	0.43
Sep	-21.46	-25.93	-4.47	-4.96	2.33	0.49
Oct	-76.81	-81.46	-4.66	-5.13	0.44	0.47
Nov	-135.23	-140.01	-4.78	-5.11	-2.19	0.33
Dec	-166.98	-171.88	-4.90	-5.30	-2.43	0.39

### 3.2 Spatial Variability

In general, *F*_*DIC*_ dominates the combined effect of rain on CO_2_ sea-air flux and the global distribution follows that of the precipitation estimates (Fig 4). However, the strongest reductions in sea-air CO_2_ flux were in higher latitudes, where *k*_*rain*_ and wet deposition combined and a cumulative reduction in sea-air flux was observed. Reductions in sea-air flux were also observed in tropical regions with high rainfall, which represent an increase in the estimated oceanic sink of CO_2_. In tropical areas with lower rainfall, an increase in net sea-air transfer was observed, decreasing the estimated oceanic sink of CO_2_ (Fig 6).

### Regional Analysis

Table 3 provides the estimated sea-air CO_2_ flux for the four regions, representing the main oceanic basins. The effect of rain on gas transfer alters the annual regional oceanic basin net integrated sea-air CO_2_ flux by -0.03 to 4.5 Tg C yr^-1^ and is primarily positive, decreasing the oceanic sink. Wet deposition alters the annual regional oceanic basin net integrated sea-air CO_2_ transfer by -2 to -32 Tg C yr^-1^, increasing the oceanic sink of CO_2_.

**Table 3:** Annual integrated net flux with rain components, *F*_*T*_ (Tg C yr^-1^) from 1999–2006, for each of the ocean basins, and the impact of each rain component on *F*_*T*_ (Tg C yr^-1^), where *F*_*T*_ = *F*_*DIC*_ + *F*_*k-rain*_ and all-rain = *F*_*T*_*−F*_*ref*_.

Year	Atlantic				Indian				Pacific				Southern			
	*F*_*T*_	All-rain	*F*_*DIC*_	*F*_*krain*_non-linear	*F*_*T*_	All-rain	*F*_*DIC*_	*F*_*k-rain*_,non-linear	*F*_*T*_	All-rain	*F*_*DIC*_	*F*_*k-rain*_,non-linear	*F*_*T*_	All-rain	*F*_*DIC*_	*F*_*k-rain*_, non-linear
99	-493	-13.2	-14.0	0.8	-355	-11.4	-12.3	0.9	-686	-28.3	-29.6	1.3	-68.1	-2.4	-2.4	-0.02
00	-504	-13.1	-13.7	0.6	-334	-11.6	-12.6	1.0	-517	-28.1	-30.4	2.3	-91.8	-2.3	-2.3	-0.03
01	-473	-12.5	-13.1	0.6	-270	-10.7	-11.6	0.9	-326	-27.3	-30.0	2.7	-16.9	-2.0	-2.0	-0.01
02	-564	-13.7	-14.2	0.6	-290	-11.4	-13.0	1.6	-198	-27.2	-31.7	4.5	-69.9	-2.3	-2.3	-0.02
03	-469	-13.5	-14.3	0.8	-322	-11.8	-13.3	1.5	-281	-27.7	-31.2	3.5	-15.9	-2.2	-2.2	-0.01
04	-447	-13.4	-14.0	0.6	-362	-11.2	-12.8	1.6	-182	-28.0	-31.6	3.6	-36.9	-1.7	-2.2	0.01
05	-385	-13.9	-14.7	0.8	-430	-12.1	-13.4	1.3	-182	-28.3	-31.3	2.9	-47.8	-2.6	-2.5	-0.01
06	-377	-13.7	-14.5	0.8	-446	-11.8	-13.2	1.4	-296	-28.1	-31.6	3.5	-33.4	-2.9	-2.9	-0.02

Regionally, during 1999 and 2000, the Pacific Ocean shows the most negative sea-air flux values, *F*_*T*_ = -686 and -517 Tg C yr^-1^, respectively (Table 3). However, subsequent years show this reducing by approximately 60% to a 2001–2006 mean of *F*_*T*_ = -244 Tg C yr^-1^, meaning that between 2002 and 2006, less CO_2_ is absorbed by the Pacific Ocean than both the Atlantic and Indian Oceans. The substantial differences between global flux during 1999–2000 and subsequent years (Fig 2 and Table 1), particularly evident in results from the Pacific (Table 3), agree with previous results [30]. The values for *p*CO_2_ have been fixed by the climatology (eq 5) and variations in overall flux estimations can be attributed to changes in wind speed and water temperature during these years. The observed differences in 1999–2000 are likely to be related to the strong La Niña event during this time.

All regions consistently show an overall reduction in annual CO_2_ flux due to rain effects, increasing the oceanic CO_2_ sink. The Pacific reduction is the strongest, varying between 5% and 15% of the total estimated flux from this region. The change in estimated CO_2_ flux in the Atlantic is approximately half the magnitude of that in the Pacific, with that in the Indian Ocean slightly less again. As such, rain effects comprise between 2.4 and 4% of annual net flux in the Atlantic and Indian ocean basins, increasing the oceanic CO_2_ sink. The Southern Ocean exhibits the smallest net change to CO_2_ flux due to rain effects. However, due to the low net total CO_2_ flux in this region, the predicted changes due to rain represent between 3% and 14% of total flux, again increasing the oceanic sink of CO_2_.

### 3.3 Temporal variability

The monthly estimated global net CO_2_ flux shows a strong, consistent seasonal cycle (Fig 2). The influence of rain is again dominated by wet deposition, *F*_*DIC*_. The global net influence of *F*_*DIC*_ varies between -5 and -5.5 Tg C month^-1^ (Table 2), representing an increase in the oceanic CO_2_ sink each month. During September, this represents 20% of the global net flux, *F*_*ref*_, reducing to 3% for December. A seasonal pattern can be observed in *F*_*DIC*_, although this is not consistent for all years.

The influence of the non-linear *k*_*rain*_ term is between 0.3 and 0.5 Tg C month^-1^ and increases net flux, which represents a decrease in the oceanic CO_2_ sink (Figs 3 and 5 and Table 1). This represents between 0.2% of total flux during December and 2% during September.

### 3.4 Errors and uncertainty

Random noise was used to perturb the input data for the year 2000, as described in section 2.5. Due to the perturbation of input signals with random noise, annual global CO_2_ flux values varied with a standard deviation of 0.7 Tg C month^-1^, taken across all 10 ensemble runs. This represents 0.7% of the estimated net integrated global flux values, which is of the same order as the 0.5% random error reported in CO_2_ sea-air fluxes in the Arctic seas [25].

### 3.5 Comparison with SOCAT Climatology

Estimations of *F*_*T*_ using both rain effects, *F*_*DIC*_ and *k*_*rain*_, were repeated with the SOCAT *pCO*_2*W*_ climatological data [14] replacing that of [13]. The SOCAT reference year is 2010 and the trend in eq 5 was applied moving back in time from 2010. As such, the two climatologies represent global pCO_2_ values for different years, adjusted for changes due to increased levels of atmospheric CO_2_, but inter-annual variability is not explicitly resolved. Furthermore, *pCO*_2_ values from Takahashi et al. [13] have been smoothed to best represent idealised non El-Niño conditions, whilst the SOCAT derived data set does not include such adjustments [14]. Estimates were made between 2004 and 2006 both with rain effects, *k*_*rain*_ and *F*_*DIC*_ and without. During this period, results using SOCAT give an average CO_2_ flux of -1600 Tg C yr^-1^ compared to -1120 Tg C yr^-1^ using [13]. Importantly for this study, the estimated effect of rain on annual net integrated CO_2_ transfer was in general agreement, with an average difference in global CO_2_ transfer of 57 Tg C yr^-1^ using [13] and 42 Tg C yr^-1^ using SOCAT. The effect of rain was to increase the oceanic sink in both cases.

## Discussion

In this work, the choice of a linear or non-linear parameterisation of the relative importance of wind and rain on gas transfer velocity is shown to have significant impact on the estimation of CO_2_ flux (Fig 3). Using a non-linear term [5], both temporal and spatial variability are diminished and the average net CO_2_ transfer is decreased. Thus, relative to the previous linear parameterisation, importance of rain for gas transfer at a global level is diminished, meaning that *F*_*DIC*_ is the more important process for the impact of rain on CO_2_ flux between the ocean and air.

This research provides a comprehensive global study into the effect of rain. However, the practicalities of capture, processing and storage of global data sets mean that it is often necessary to compromise on spatial and/or temporal resolution. In the case of the rain data from GPCP, the global data set is available as monthly averages in mm day^-1^, averaged spatially over 1^°^ x 1^°^. At these scales, it is not possible to resolve intense episodic or extreme events. This raises three areas for consideration. Firstly, the transfer velocity during a single day of heavy rain within a month will not be equivalent to that calculated using eq 9, based on a monthly average rain rate. Secondly, as discussed above, the lack of knowledge of actual rain rates during these episodes will prevent the direct estimation of the extent of temporary surface dilution and its impact on gas exchange. Finally, correlation between wind and rain within the month will also affect the gas exchange and again, cannot be predicted.

Taking these three areas in turn, the first is surface dilution. Rain falling onto the ocean will influence the chemical properties of surface waters. As such, it could decrease the *pCO*_2*W*_ and directly affect CO_2_ exchange. Observational studies from Biosphere ocean experiments provide evidence for the formation of freshwater layers [3, 4]. There is also in-situ evidence from the Pacific region, with direct measurements of decreased salinity at the surface during and after rain events [31]. These sources identify a peak in the freshwater layer after approximately 1 hour of persistent rain and highlight changes in surface stratification up to two days after the rain event.

Experimental data to estimate the effect of surface dilution on CO_2_ exchange exist and Turk et al. [7] consider dilution for a point in the Western Equatorial Pacific. The same temporary changes to surface water composition have been seen to affect remotely-sensed salinity measurements [8] and methods have been proposed to relate these to rain rate [9], or use physical modeling to predict their existence, in order to better understand variability in remote sensing data. In theory, such methods could be applied to predict the impact of freshwater layers on gas exchange. However, the spatial and temporal scales of the estimates made here are limited by the global data sets and are not sufficient to resolve individual events.

In addition to chemical dilution, rain falling on the sea surface could affect *SST*. Gas solubility is a function of salinity and temperature and changing *SST* will affect the CO_2_ balance across the surface, altering exchange between air and atmosphere through eqs 11 and 12. The high temperature dependency of *pCO*_2*W*_ suggests that this could be an important process to consider [32, 33]. Gosnell et al. [34] used a modeling study to investigate the relative temperature of the rain to the sea surface through estimations of the changing temperature of raindrops. In their experiment, a maximum 0.2K difference occurs at maximum rain rates (100 mm hr^-1^) from maximum height (5000 m). As an initial investigation, temperature differences were applied to the *SST* input data for the reference year 2000. A constant bias of 0.2 K was subtracted from surface *SST* for calculations where the rain rate exceeds 1 mm hr^-1^. The observed differences were negligible, resulting in the flux being altered (reduced or increased) by up to 0.02% of monthly regional integrated net CO_2_ flux. These results imply that the rain-induced temperature differences have a negligible effect on the air-sea gas fluxes and significantly less than the total uncertainties (0.7%) calculated in section 3.3.

Global rain rate retrievable through the GPCP is the monthly average for a 1^°^ x 1^°^ grid square. Here, this has been used in eqs 8, 9 & 10 to calculate the gas transfer velocity, *k* as a combination of wind, *k*_*wind*_ and rain, *k*_*rain*_, assuming constant and consistent rain rate throughout the area and throughout the month. In reality, the rain will fall at varying rates during a month and within a grid square, which will cause variability in *k*_*rain*_, as well as the ratio of kinetic energy flux between wind and rain, *β*, which governs the contribution of *k*_*rain*_. Heavy rain for two days in a month and no other rain, will not affect *k*_*total*_ by the same magnitude as the same rain spread over the month. However, *Rn* used in these studies will be the same and the temporal (or spatial) variability cannot be accounted for. In order to examine how this will affect the overall outcome, the [1*-* exp(*-aβ*)] *k*_*rain*_ term in eq 10 was calculated for an example average monthly rain rate of 1mm/hr spread over a varying number of days in a month. At low wind speeds (u < 10m s^-1^), spreading the rain over the month (as is assumed with a monthly mean) gives a higher estimate for *k*_*rain*_, and subsequently, *k*_*total*_, than shorter duration heavier rain. However, at higher wind-speeds (*u* > 10 m s^-1^), the *β* ratio means that low rain rates are estimated to have little effect. As such, the shorter duration, heavier rain produces a higher estimate for the influence of rain on *k*_*total*_. This means that, the methodology may be under-estimating the effect of rain in higher latitudes and overestimating in lower latitudes. Nevertheless, *F*_*k-rain*_ has an impact on the overall flux that is a factor of 10 smaller than that of direct deposition, *F*_*DIC*_, limiting the overall impact of variability on global results. When examining regionally, the effect will become more important and in the future, more detailed data for the pattern of rainfall would be beneficial, particularly to small regional studies in areas where *F*_*k-rain*_ is relatively important, such as those with high rainfall and low wind speeds..

Within a grid-square and during a month, there will also be variability in the wind strength. It is a combination of wind and rain rate will govern the extent and duration of surface dilution, as well as the effect of temporal and spatial variability. Typically, in mid-latitudes, rain events are associated with developing low pressure systems and so correspond with stronger winds than average for a region. In tropical latitudes, precipitation occurs both in storms and in large convective systems with relatively gentle low-level convergence (Plate 4 of Quartly et al. [35]). More recently, Quartly et al. [36] confirmed the predominance of rain at low wave heights for a number of regions in the Atlantic, with, for some seasons, rainfall in mid-latitudes being roughly five times as likely at low sea state than at high. Further work should look towards measuring the instantaneous relationship between wind and rain. There are a number of different remote-sensing technologies that can make estimates of the rain rate at the Earth's surface. Dual-frequency altimeters can provide simultaneous estimates of wind speed, wave height and rain rate. These could support studies of the correlation of these conditions [35], or even direct measurement of the *β* ratio

It must also be noted that here we have assumed that the GPCP precipitation data characterises only rainfall, whereas precipitation also includes sleet, ice and snow. However, we have no information on how much snow and sleet falls globally each year so the impact of this assumption is unknown.

## Conclusions

This paper has presented analysis of the impact of rain on global and regional sea-air CO_2_ fluxes and the oceanic net sink of CO_2_. The work has exploited the open source FluxEngine software, cloud computing, advanced methods for estimating rain induced sea-air gas fluxes and an extensive dataset of climate quality satellite Earth observation, in situ, model and re-analysis data.

The results demonstrate a non-negligible effect of rain when estimating global and regional integrated net sea-air CO_2_ fluxes. Differences of approximately 6% in annual global CO_2_ flux have been estimated, which means that rain serves to increase the oceanic CO_2_ sink.

Implementing the non-linear relationship between rain and wind, as recommended by Harrison et al. [5], over the linear relationship originally proposed by Ho et al. [12], significantly reduces the spatial and temporal variability with which rain enhancement of gas transfer rate affects CO_2_ flux. This serves to diminish the importance of this rain induced gas transfer in the effect of rain on integrated net sea-air CO_2_ fluxes

Globally, the observed changes are dominated by the influence of wet deposition, *F*_*DIC*_. The influence of rain varies regionally and is greatest in the Pacific Ocean where it represents up to 15% of the annual regional net flux, and up to 50% of monthly net flux. It is also important in the Southern Ocean, due to the low overall CO_2_ sink estimate, where it represents 13% of annual net flux. Regional fluxes are more variable, with up to 16% modulation of the annual integrated net CO_2_ flux due to rain, which can be responsible for turning the region from a net source to a net sink.

Therefore we conclude that the impacts of rain should be included in the uncertainty analysis of studies that estimate integrated net sea-air fluxes of CO_2_. However, for regional or short-term studies, results suggest that rain can have a considerable impact on the fluxes, dependent upon the region and timescale and may need to be considered directly in sea-air CO_2_ flux estimates.

Three key limitations of current global datasets for deriving more accurate measures of the effect of rain on gas transfer have been highlighted. Further work to exploit con-incidental wind and rain data sets and associated development of a generalised parameterisation relating wind and rain rate to the concentration balance of trace gases across the interface offers significant potential in this area.
